# Supplementary material for: Splice-Junction-Based Mapping of Alternative Isoforms in the Human Proteome
Source: Cell Rep. Author manuscript; Available in PMC 2020 Jan 15. (PMC6961840; doi:10.1016/j.celrep.2019.11.026)
Supplement: 3 [file NIHMS1546469-supplement-3.zip › DF2/PXD0010154/Ovary-130-P27816-MYHDDDLADLVFPSSATADTSIFAGQNDPLKDSYVPLELAK.pdf]

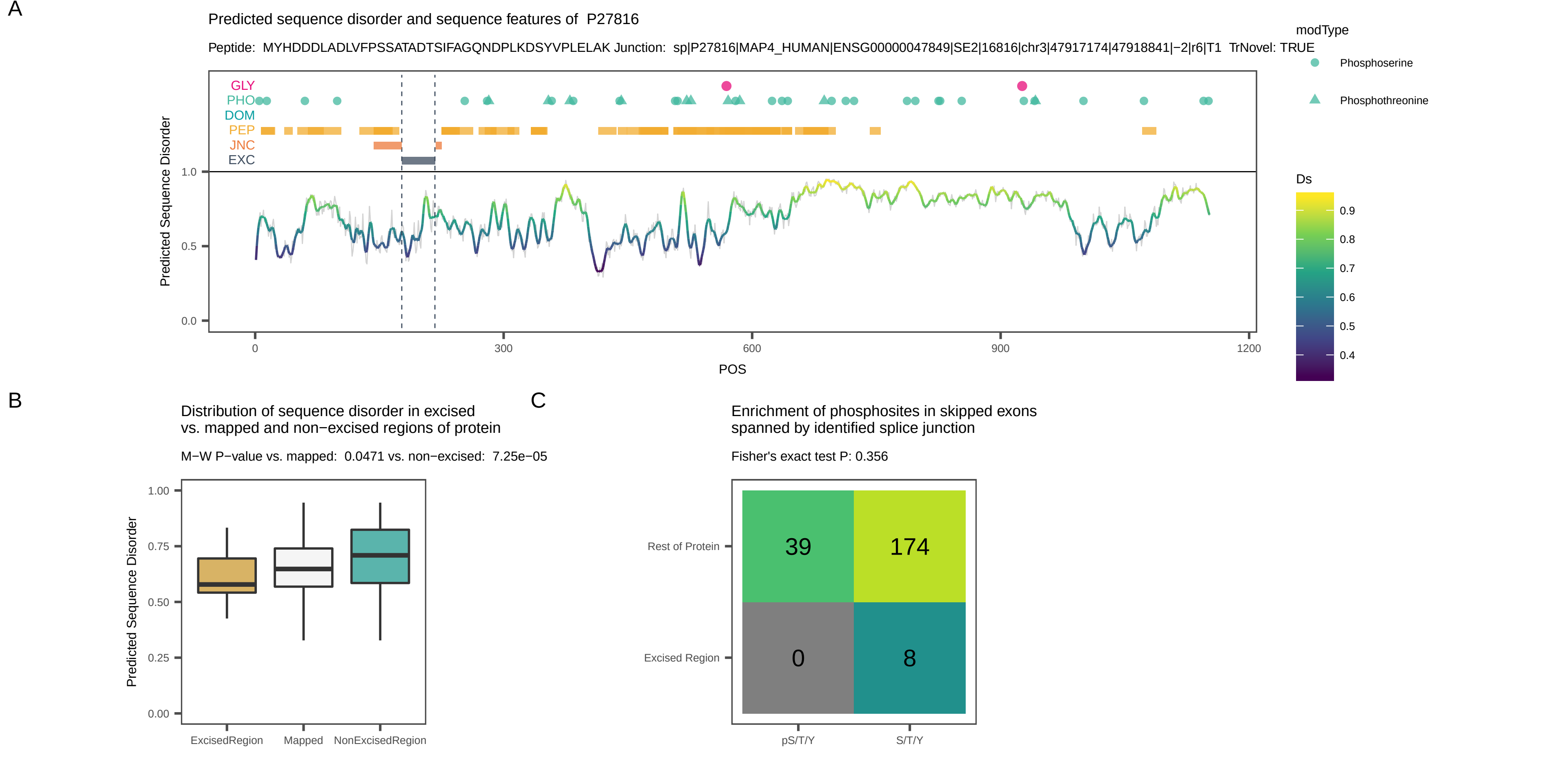
A
Predicted sequence disorder and sequence features of P27816
Peptide: MYHDDDLADLVFPSSATADTSIFAGQNDPLKDSYVPLELAK Junction: sp|P27816|MAP4_HUMAN|ENSG00000047849|SE2|16816|chr3|47917174|47918841|−2|r6|T1 TrNovel: TRUE
modType
Phosphoserine
Phosphothreonine
GLY
PHO
DOM
PEP
JNC
EXC
Predicted Sequence Disorder
1.0
0.5
0.0
0
300
600
900
1200
POS
Ds
0.9
0.8
0.7
0.6
0.5
0.4
B
Distribution of sequence disorder in excised vs. mapped and non−excised regions of protein
M−W P−value vs. mapped: 0.0471 vs. non−excised: 7.25e−05
Predicted Sequence Disorder
1.00
0.75
0.50
0.25
0.00
ExcisedRegion
Mapped
NonExcisedRegion
C
Enrichment of phosphosites in skipped exons spanned by identified splice junction
Fisher's exact test P: 0.356
Rest of Protein
Excised Region
39
174
0
8
pS/T/Y
S/T/Y
